# Characterizing the bacterial communities associated with Mediterranean sponges: a metataxonomic analysis

**DOI:** 10.3389/fmicb.2023.1295459

**Published:** 2024-01-11

**Authors:** Roberta Esposito, Serena Federico, Michele Sonnessa, Sofia Reddel, Marco Bertolino, Nadia Ruocco, Giacomo Zagami, Marco Giovine, Marina Pozzolini, Marco Guida, Valerio Zupo, Maria Costantini

**Affiliations:** ^1^Department of Ecosustainable Marine Biotechnology, Stazione Zoologica Anton Dohrn, Napoli, Italy; ^2^Department of Earth, Environmental and Life Sciences, University of Genoa, Genoa, Italy; ^3^Bio-Fab Research Srl, Rome, Italy; ^4^Department of Ecosustainable Marine Biotechnology, Stazione Zoologica Anton Dohrn, Calabria Marine Centre, Amendolara, Italy; ^5^Dipartimento Di Scienze Biologiche, Chimiche, Farmaceutiche Ed Ambientali, Università Di Messina, Messina, Italy; ^6^Department of Biology, University of Naples Federico II, Complesso Universitario di Monte Sant'Angelo, Naples, Italy; ^7^Department of Ecosustainable Marine Biotechnology, Stazione Zoologica Anton Dohrn, Ischia Marine Centre, Naples, Italy

**Keywords:** bacteria, metataxonomic analysis, molecular identification, morphological identification, sponges

## Abstract

The oceans cover over 70% of our planet, hosting a biodiversity of tremendous wealth. Sponges are one of the major ecosystem engineers on the seafloor, providing a habitat for a wide variety of species to be considered a good source of bioactive compounds. In this study, a metataxonomic approach was employed to describe the bacterial communities of the sponges collected from Faro Lake (Sicily) and Porto Paone (Gulf of Naples). Morphological analysis and amplification of the conserved molecular markers, including 18S and 28S (RNA ribosomal genes), CO1 (mitochondrial cytochrome oxidase subunit 1), and ITS (internal transcribed spacer), allowed the identification of four sponges. Metataxonomic analysis of sponges revealed a large number of amplicon sequence variants (ASVs) belonging to the phyla Proteobacteria, Cloroflexi, Dadabacteria, and Poribacteria. In particular, *Myxilla* (*Myxilla*) *rosacea* and *Clathria* (*Clathria*) *toxivaria* displayed several classes such as Alphaproteobacteria, Dehalococcoidia, Gammaproteobacteria, Cyanobacteria, and Bacteroidia. On the other hand, the sponges *Ircinia oros* and *Cacospongia mollior* hosted bacteria belonging to the classes Dadabacteriia, Anaerolineae, Acidimicrobiia, Nitrospiria, and Poribacteria. Moreover, for the first time, the presence of Rhizobiaceae bacteria was revealed in the sponge *M*. (*Myxilla*) *rosacea*, which was mainly associated with soil and plants and involved in biological nitrogen fixation.

## 1 Introduction

The Mediterranean Sea is considered very significant in terms of biodiversity, as it hosts 7% of the world's marine biodiversity (with only 0.82% of the oceans' surface), including a large number of endemic species, which are also subject to anthropogenic stress destined to increase in the future (Coll et al., 2010). Among marine organisms, marine sponges emerged on the earth more than 600 million years ago (the Cambrian Age) and represented one of the most important components of benthic fauna. They are extensively distributed from polar to tropical waters and from intertidal regions to waters thousands of meters deep (Fusetani, 1996), representing dominant taxon in marine communities in terms of species biomass, richness, and spatial coverage (Corriero et al., 2000). A miscellaneous of archaea, heterotrophic bacteria, cyanobacteria, green algae, red algae, cryptophytes, dinoflagellates, and diatoms have been found together with sponges (Larkum et al., 1987; Santavy et al., 1990; Duglas, 1994). Their microbial community is very diverse, with species composition showing temporal and geographical differences (Webster and Hill, 2001). A sponge can be associated with diverse organisms, such as *Theonella swinhoei*, which hosted unicellular bacteria and cyanobacteria at the same time (Bewley et al., 1996). Additionally, a sponge belonging to the *Aplysina* genus included heterogeneous bacteria *Micrococcus* sp., *Bacillus* sp., *Arthrobacter* sp., *Pseudoalteromonas* sp., *Vibrio* sp., and others (Hentschel et al., 2001). The surfaces or internal sides of marine sponges are richer in nutrients than seawater and sediments; therefore, sponges offer both a feeding source and a secure habitat for their microorganisms (Bultel-Poncé et al., 1999). On the other hand, symbiotic microorganisms contribute to the nutritional process through intracellular digestion and/or translocation of metabolites, such as nitrogen fixation, nitrification, and photosynthesis (Wilkinson and Fay, 1979; Wilkinson and Garrone, 1980). Microorganisms also stabilize the skeleton of sponges (Wilkinson et al., 1981) and participate in the host chemical defense system against hunters and biofouling (Bakus et al., 1986; Paul, 1992; Proksch, 1994; Unson et al., 1994). Sponges and associated microorganisms represent very useful organisms as sources of bioactive compounds for marine biotechnology (Cooper and Yao, 2010; Karuppiah and Li, 2015; Thompson et al., 2017; Cerrano et al., 2022). In fact, one of the first peptides with antibacterial activity was isolated by the gram-negative bacterium *Vibrio* sp. associated with the sponge *Hyatella* sp. (Oclarit et al., 1994). Starting from this, several studies showed pharmacological applications of natural products from marine sponges and their associated biota (Newman and Cragg, 2020; Zhang et al., 2020; Amelia et al., 2022).

In the present study, we deeply explored the bacterial communities associated with four sponges collected in the Mediterranean Sea. These species were collected in Italy in two different areas: Faro Lake (Sicily) and Porto Paone (Gulf of Naples). Species were characterized by skeletal morphological observation and amplification of several conserved molecular markers, represented by RNA ribosomal genes (*18S* and *28S rRNA*), mitochondrial cytochrome oxidase subunit 1 (*CO1*), and internal transcribed spacer (*ITS*). To analyze the biodiversity of symbiotic communities between the two sampling sites, we performed metataxonomic analysis of sponge samples using the Illumina MiSeq platform. More than 1,000 bacterial isolates from four samples were phylogenetically identified to understand if they are host-specific and/or location-specific. Subsequently, the ASV analysis was also applied to reveal the level of variability in the host-specific microbial communities, which were then discussed to assess the biotechnological potential of the sponges under analysis.

## 2 Materials and methods

### 2.1 Sponge sampling

Two sponge samples (indicated as S36 and S39) were collected in the Gulf of Naples by scuba divers from the Stazione Zoologica Anton Dohrn. The sampling site was Porto Paone (40°47′N, 14°9′E), and the specimens were collected at depths between 15 and 17 m. The other two sponges (indicated as S23 and S25) were collected at the Faro Lake in Messina, Sicily (38°16′N, 15°38′E) at depths between 2 and 3 m (Ruocco et al., 2021, see Table 1). This sampling site is located in the Natural Reserve of “Capo Peloro.” The Faro Lake has the shape of a funnel, and due toits maximum depth of 29 m, which more or less coincides with the center of the lake, it is considered the deepest coastal lake in Italy. The Faro Lake is a salty lake connected by two channels to the Strait of Messina and the Tyrrhenian Sea (northern and northeastern side). The abiotic lake parameters of the lake include a salinity range of 26–36 PSU, pH levels ranging from 7.0 to 8.6, and temperature ranging from 10 to 30°C (Saccà et al., 2008; Marra et al., 2016).

**Table 1:** Sample IDs, taxonomy, sampling depth (m), sites, geographical coordinates, and type of substrate of sponge specimens.

**Sample IDs**	**Sponge taxonomy**	**Sampling depth (m)**	**Sampling site**	**Coordinates**	**Substrate**
S23	*Clathria* (*Clathria*) *toxivaria*	2–3	Faro Lake	38°16′N, 15°38′E	Rocks, coralligenous concretions, caves, and epibiotic on other organisms
S25	*Myxilla* (*Myxilla*) *rosacea*	2–3	Faro Lake	38°16′N, 15°38′E	Rocks, coralligenous concretions, caves, and epibiotic on other organisms
S36	*Ircinia oros*	15–17	Porto Paone	40°47′N, 14°9′E	Sand, mud, rocks, coralligenous concretions, and cave
S39	*Cacospongia mollior*	15–17	Porto Paone	40°47′N, 14°9′E	Sand, mud, rocks, coralligenous concretions, cave, and epibiotic on other organisms

### 2.2 Morphological identification of the sponges

The taxonomic analysis was conducted by examining the skeletal architecture and spicule complement of each sponge specimen according to the protocols already published (Núñez-Pons et al., 2022). The taxonomic identification was made at species level as described in the World Porifera Database (WPD; Van Soest et al., 2023).

### 2.3 Molecular identification of sponges

The DNA extraction was performed starting from 10 mg of sponge tissue and employing QIAamp^®^ DNA Micro kit (QIAGEN, Germany), according to the manufacturer's protocol. The DNA (ng/μL) was quantified using NanoDrop spectrophotometer. The C1000 Touch Thermal Cycler (Biorad) was used for PCR reaction processes in a 30 μL final volume of master mix with ~50–100 ng of genomic DNA. The reaction mixture was prepared using 6 μL of 5X Buffer GL (GeneSpin Srl, Milan, Italy), 3 μL of dNTPs (2 mM each), 2 μL each of forward and reverse primers (25 pmol/μL), and 0.5 μL of Xtra Taq Polymerase (5 U/μL, GeneSpin Srl, Milan, Italy) as follows:

- For 18S and 28S, the amplification conditions were 95°C for 2 min followed by 35 cycles of 95°C for 1 min, 60°C (A/B) and 55°C (18S-AF/18S-BR, NL4F/NL4R) for 1 min, and 72°C for 2 min, with a final extension step at 72°C for 10 min (Chombard et al., 1998; Manuel et al., 2003);
- ITS primers (RA2/ITS2.2), with initial denaturation at 95°C for 2 min. This was followed by 35 cycles of 95°C for 1 min, 67°C for 1 min, and 72°C for 2 min, with a final extension step at 72°C for 10 min (Worheide et al., 2002; Schmitt et al., 2005);
- CO1 primers (dgLCO1490/dgHCO2198), with initial denaturation at 94°C for 3 min, 35 cycles of denaturation at 94°C for 30 s, 45°C for 30 s, and a final step of elongation of 72°C for 1 min (Meyer et al., 2005).

The gel of agarose (1.5% electrophoresis in 40 mM Tris–acetate, 1 mM EDTA, pH 8.0, TAE buffer) was used to separate PCR products; the amplified fragments were identified using a DNA ladder of 100 bp (GeneSpin Srl, Milan, Italy). The QIAquick Gel Extraction Kit (Qiagen) was used to extract the fragments from the gel following the instructions of the manufacturer. The NanoDrop spectrophotometer evaluated the DNA quantity (ng/μL). Applied Biosystems (Life Technologies) 3730 Analyzer (48 capillaries) sequenced both strands of the obtained amplicons. All the sequences from PCR products were then aligned to GeneBank using BLAST (Basic Local Alignment Search Tool) to reach for high similarity sequences. Then, Multialign was used to confirm the alignment with high similarity sequences (http://multalin.toulouse.inra.fr/multalin/).

### 2.4 Metagenomic DNA extraction, illumina miseq sequencing, and diversity analysis

The DNeasy^®^ PowerSoil^®^ Pro Kit (QIAGEN) was used for the extraction of DNA following the protocol described by the manufacturer. For this extraction, a piece of the tissue weighing about 250 mg was used. Using the NanoDrop spectrophotometer it was possible to evaluate DNA quantity (ng/μL) and quality (A260/280, A260/230). To check for DNA integrity, the samples were loaded on 0.8% agarose gel electrophoresis immersed in TAE buffer. For metataxonomic analysis performed by Bio-Fab Research (Roma, Italy), a final concentration of 30 ng/μL of DNA was used. Illumina adapter overhang nucleotide sequences were added to the gene-specific primer pairs targeting the V3-V4 region (S-DBact-0341-b-S-17/S-D-Bact-0785-a-A-2), according to Klindworth et al. (2013). 16S PCR amplification was performed in a final volume of 25 μL, which was set up using the following quantities: 5 μL of microbial genomic DNA (10 ng/μL in 10 mM Tris pH 8.5), 1x PCRBIO HiFi Buffer (PCR BIOSYSTEMS, USA) composed by 1 mM of dNTPs and 3 mM of MgCl_2_, 0.5 units of PCRBIO HiFi Polymerase (PCR BIOSYSTEMS, USA), and 0.2 μM of each primer. Cycling conditions followed an initial denaturation at 95°C for 3 min, 25 cycles of 95°C for 30 s, 55°C for 30 s, 72°C for 30 s and a final extension step at 72°C for 5 min, hold at 4°C. After the amplification of the 16S, a PCR clean-up was done to purify the V3-V4 amplicon from free primers and primer dimers. After that, a limited-cycle amplification step using Nextera XT Index Kit was carried out to add multiplexing indices and illumine sequencing adapters. In addition, a further clean-up step was performed, followed by a normalization of the libraries and their pooling by denoizing processes. The sequencing on Illumina MiSeq Platform with 2×300 bp paired-end reads took place only after these steps were completed. Taxonomy assignments were done using a “home-made” Naive Bayesian Classifier trained on V3-V4 16S sequences of the SILVA 138.1 release database (Quast et al., 2013). The sample frequencies per feature and per sample are indicated in Supplementary Figures 1, 2. The metataxonomic analysis from raw DNA sequencing data was conducted on the Quantitative Insights Into Microbial Ecology (QIIME 2, Version: 2022.2) platform (Bolyen et al., 2019) by demultiplexing, quality filtering, chimera removal, taxonomic assignment, and both alpha and beta diversity analyses. With the use of R version 4.1.1 (Li, 2021) and Cairo graphics library (Urbanek and Horner, 2020), the taxonomy barplot was generated. To evaluate the species diversity, three different indices were took into account: Chao 1 index (Chao, 1984) is a qualitative species-based method (species richness in the sample that is the number of ASV) and Shannon (1948), Shannon and Weaver (1949), and Simpson (1949) indexes estimated the quantitative species-based measures, which indicated the community diversity as species richness and evenness. The estimation was made at three taxa levels (Level 5 = Family, Level 6 = Genus, Level 7 = Species). Statistically significant differences for alpha and beta diversities were explored using the Kruskal–Wallis test and pairwise PERMANOVA analysis, respectively. The distance matrix between each pair of sample (independently from environmental variables) was calculated using Bray–Curtis and “un-, weighted” UniFrac metrics.

The data were deposited in the SRA database (submission ID: SUB14019148; BioProject ID: PRJNA1049642).

## 3 Results

### 3.1 Morphological and molecular characterization

All the sponges were characterized by morphological and molecular approaches. Concerning the morphological analysis, all the analyzed sponges belonged to the class Demospongiae (see Table 1) and usually live in the Mediterranean Sea. Concerning *C*. (*Clathria*) *toxivaria*, collected in the Faro Lake (Sicily), molecular tool for characterization of this species identified by morphological analysis was not available in GenBank. The second sponge collected in the Faro Lake was identified by morphological analysis as *M*. (*Myxilla*) *rosacea*. In the case of the two sponge samples collected in “Porto Paone” (Gulf of Naples), CO1 was the best molecular marker with 98.5% identity, characterizing the sponge S36 as *Ircinia oros* with 98.5% identity. For Sample S39, this species was identified by ITS as *Cacospongia mollior*, reporting a percentage of sequence similarity of 99%.

### 3.2 Metataxonomic profile

**Figure 1:**
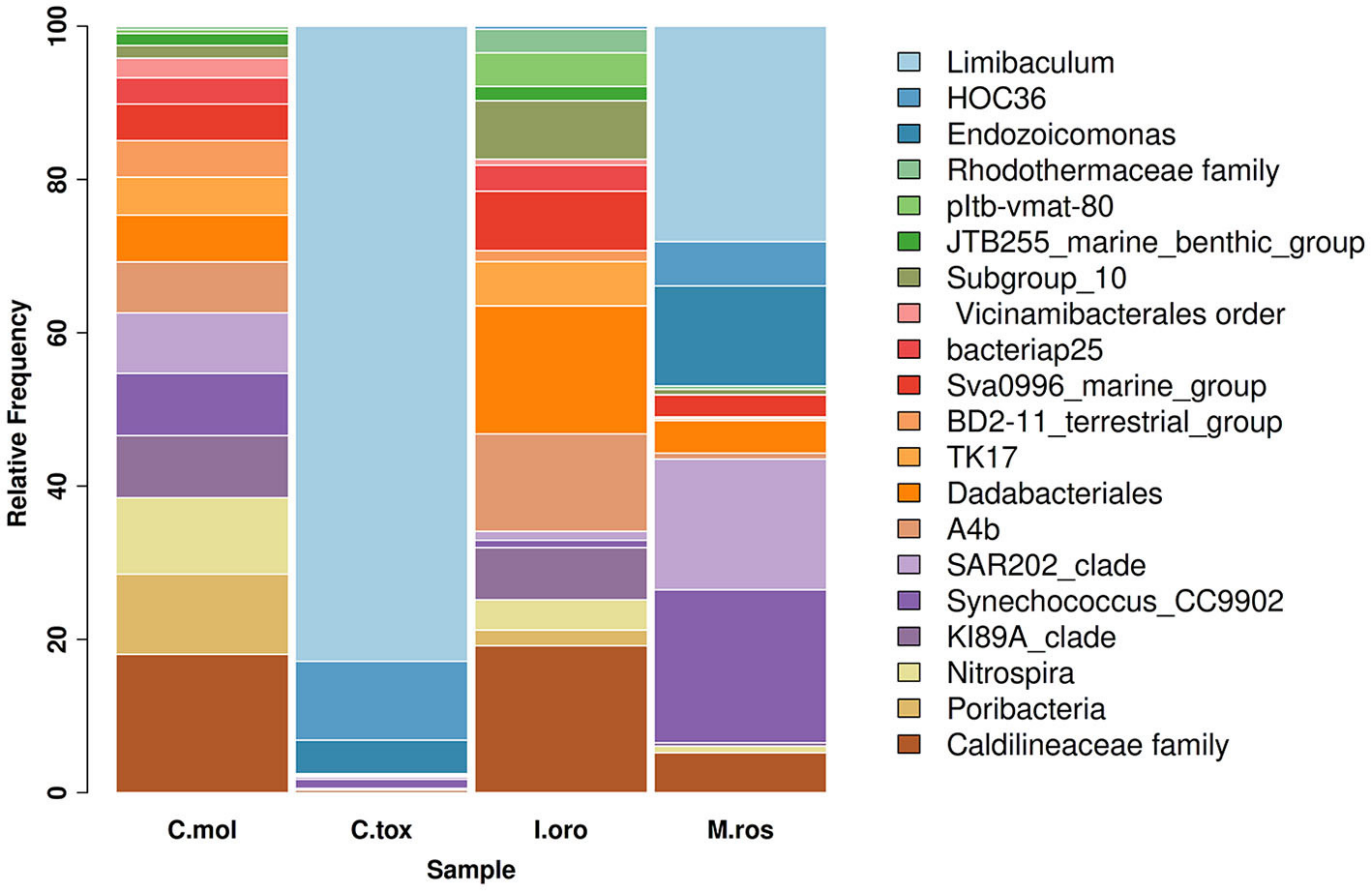
Taxonomy barplot comparing the most abundant bacterial phyla at the genus level in sponges *C. mollior, C*. (*C*.) *toxivaria, I. oros*, and *M*. (*M*.) *rosacea*. Data were normalized in each sample by median sequencing depth, filtered to low abundance and to sum rows >500.

As indicated by the ASVs analysis, most microbial species had a confidence percentage ≥75%. The complete taxonomy classification of bacterial communities of sponge samples is presented in Supplementary Tables 1–4. In Porto Paone, two sponges (Gulf of Naples) were found: (i) *M. (M.) rosacea* showed the largest number of features (519), especially Alphaproteobacteria, Dehalococcoidia, Gammaproteobacteria, and Cyanobacteria (Figure 1) and (ii) 250 ASVs in *C*. (*C*.) *toxivaria*, with a greater abundance of Alphaproteobacteria, Gammaproteobacteria, and Bacteroidia (Figure 1). In sponges *I. oros* and *C. mollior*, both collected in the Faro Lake (Sicily), 156 and 204 ASVs were detected, respectively. The most abundant bacterial classes in *I. oros* were Dadabacteriia, Anaerolineae, and Acidimicrobiia, while the bacterial community of *C. mollior* was dominated by Nitrospiria, Anaerolineae, Dadabacteriia, and Poribacteria (Figure 1). In particular, the taxonomic composition revealed an abundance of Proteobacteria present in *C. toxivaria* (36%) and *M*. (*M*.) *rosacea* (35%; Figure 1). In contrast, a low percentage (2%) of Proteobacteria was detected in two sponges collected from the Faro Lake (Sicily). In addition, *C. toxivaria* revealed 1% of Bacteroidia class. The sponge *M*. (*M*.) *rosacea* revealed low percentages of Cyanobacteria and Dehalococcoidia (1–0.9%, respectively; Figure 1). Interestingly, this sponge was the only species revealing a certain abundance of Rhizobiaceae, belonging to the phylum Proteobacteria (75.6 %; Figure 1). Concerning the sponges *I. oros* and *C. mollior*, low percentages of Dadabacteria (1%) and Poribacteria (0.9%) were present, respectively (Figure 1). As reported above, the sponges *C*. (*C*.) *toxivaria* and *M*. (*M*.) *rosacea* revealed a comparable composition in the distribution of microbial species. In fact, a great plethora of Proteobacteria was observed in these species (Figure 1). Similarly, the sponges *I. oros* and *C. mollior* showed the same distribution of bacteria and archaea.

### 3.3 Principal coordinates analyses (PCoA) of bacterial communities

**Figure 2:**
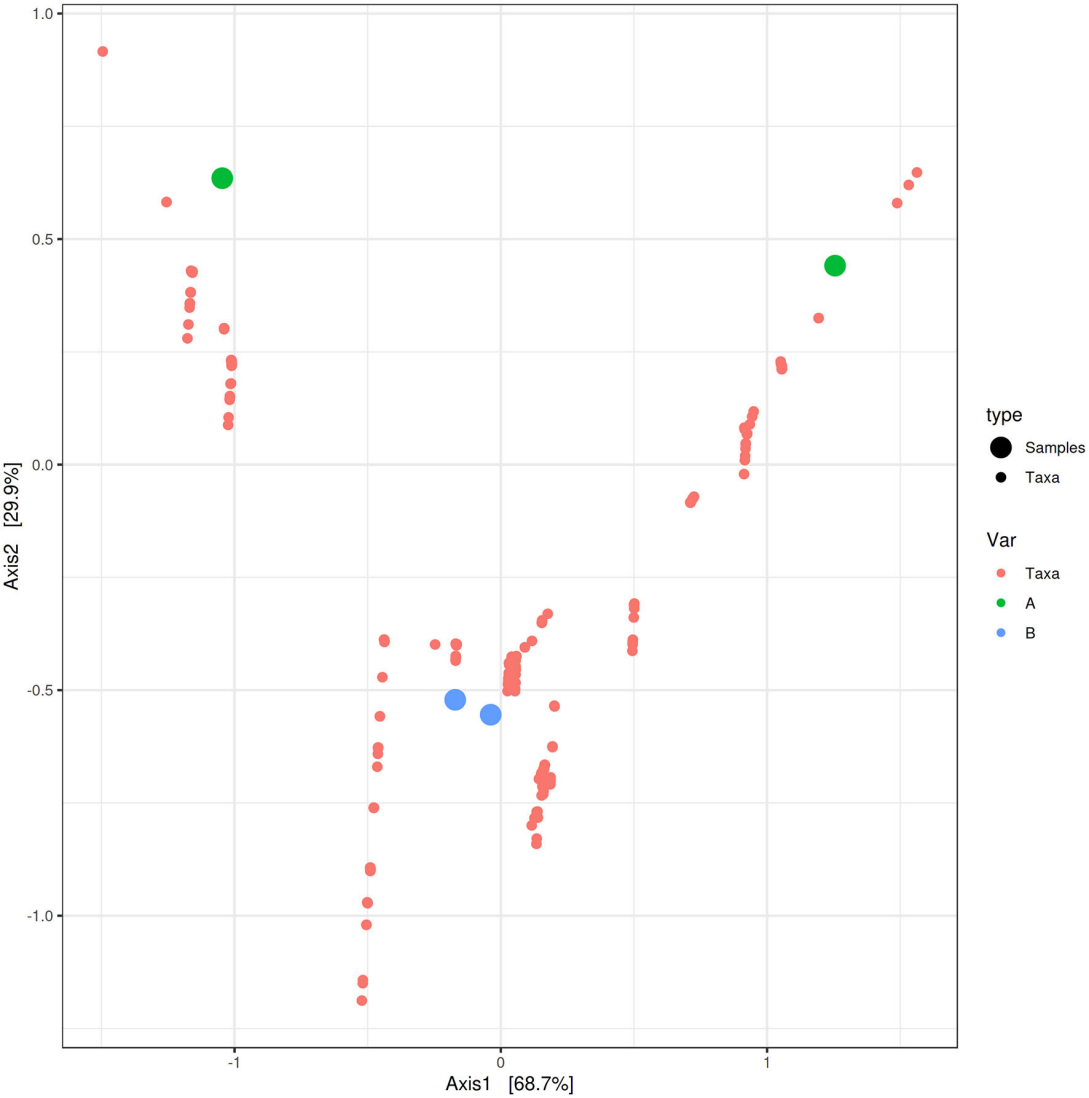
Principal coordinates analysis (PCoA) biplot between Group A and Group B for identifying features that contribute the most in terms of separating the samples in a PCoA plot.

**Figure 3:**
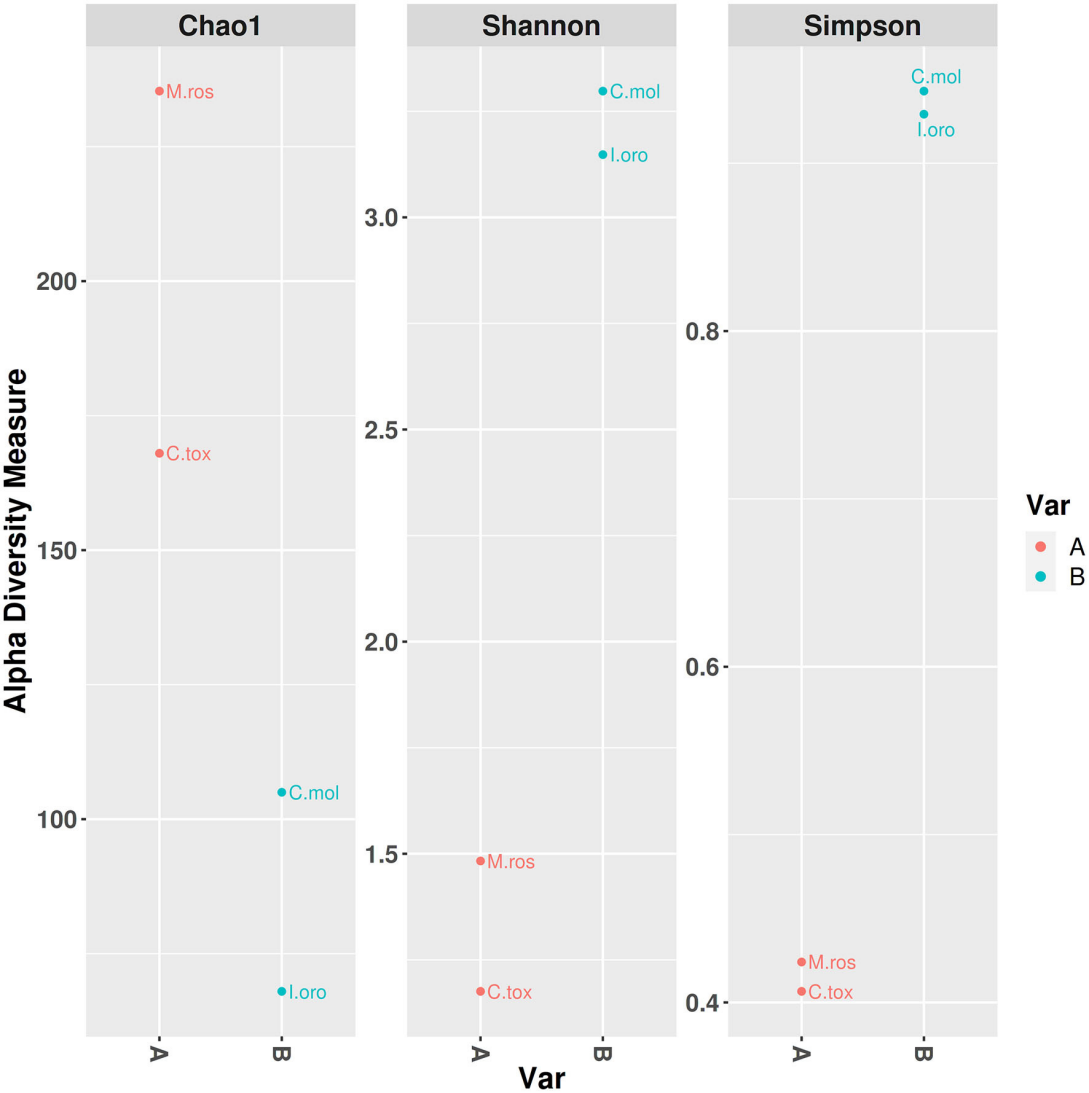
Alpha diversity plot with separate variable A and B.

PCoA based on the relative abundance of genera revealed a significant separation in bacterial community composition between Group A (sponges collected in the Faro Lake) and Group B (sponges collected in Porto Paone), using both principal component scores of PC1 and PC2 (68.7 and 29.9%, respectively; Figure 2 and Table 2). Alpha diversity was used to analyze the samples, and three diversity indices (Chao1, Shannon, and Simpson) were used to determine whether each sample was sequenced at sufficient depth or not. As shown in Figure 3, the Chao index of Group B, including sponges collected in Porto Paone, was lower than that of the sponges collected in the Faro Lake (Group A). The results showed that the species richness of Group B was significantly lower than that of Group A. Moreover, Shannon and Simpson indexes of the Group B were higher than that of the Group A, demonstrating that the two groups had the same trend.

**Table 2:** Bacteria clustered with *C. toxivaria, M. rosacea, I. oros*, and *C. mollior* identified at genus, family, and order levels.

	***C. toxivaria* (Axis 1 1.25528583537352 and Axis 2 0.441014958183311)**	***M. rosacea* (Axis 1 −1.04618839398916 and Axis 2 0.634895116498213)**	***I. oros* (Axis 1 −0.0380512968287544 and Axis 2 −0.55442518705781)**	***C. mollior* (Axis 1 −0.171355948499607 and Axis 2 −0.521374123467597)**
Genus	DEV007	Albidovulu	A4b	AncK6
	Endozoicomonas	AT-s3-44	bacteriap25	Aquimarina
	Entotheonellaceae	Constrictibacter	BD2-11_terrestrial_group	Blastocatella
	Filomicrobium	Cyanobium_PCC-6307	Candidatus_Tenderia	Candidatus_Kaiserbacteria
	JTB255_marine_benthic_group	EF100-94H03	EC94	Candidatus_Nitrosopumilus
	Limibaculum	JG30-KF-CM66	Exiguobacterium	FS142-36B-02
	NS5_marinegroup	Nitrospira	HOC36	Ga0077536
	Pseudohongiella	PAUC43f_marine_benthic_group	NS4_marine_group	NB1-j
	Roseibacillus	Poribacteria	Planctomicrobium	PAUC26f
		Subgroup_11	pltb-vmat-80	Rhodopirellula
		Vibrio	Ruegeria	Saccharimonadales
			Shewanella	Subgroup_9
Genus			Thalassotalea	Subgroup_21
			Turneriella	TK17
			UBA10353_marine_group	TK30
				Woeseia
Family	Clostridiaceae	Kiloniellaceae	Bacteriovoraceceae	Magnetospiraceae
		Spirochaetacea	Rhizobiaceae	Rhodobacteracea
			Rhodothermacea	Thiotrichaceae
Order				Caldilineales
				Defluviicoccales
				Vicinamibacterales

## 4 Discussion

This study builds upon previous investigations where we analyzed the microbial diversity by metataxonomic analysis of eight sponge samples from four sites: *Oceanapia* cf. *perforate* (Sarà 1960), *Sarcotragus spinosulus* (Schmidt 1862) and *Erylus discophorus* (Schmidt 1862) from Faro Lake in Sicily; *Tethya aurantium* (Pallas 1766) and *Axinella damicornis* (Esper 1794) from “Porto Paone” (Gulf of Naples); *Geodia cydonium* (Jamenson 1811) from “Secca delle fumose” (Gulf of Naples); *Agelas oroides* (Schmidt 1864) and *Acanthella acuta* (Schmidt 1862) from “Punta San Pancrazio” (Gulf of Naples). In fact, here we explored the sponge biodiversity, in terms of microorganisms, in the Mediterranean Sea, analyzing other species from the Strait of Messina and the Gulf of Naples.

We first identified four sponges, combining the identification by morphological features with a molecular approach, based on the sequences of *28S* and *18S rDNA, ITS*, and *CO1*. According to the literature, there is no single marker for all sponge species, and each primer pair displayed its own constraints and strengths (Yang et al., 2017; Ruocco et al., 2021). The difficulty in identifying them is also related to absent or incomplete sequences annotated in the database, thus limiting the phylogenetically based taxonomic methods used for species identification. For this reason, a molecular approach with multi-loci should be employed to ensure reliable sponge identification.

This represents the first record of metataxonomic analysis for the sponges, such as *C*. (*C*.) *toxivaria, M*. (*M*.) *rosacea, Ircinia oros*, and *Cacospongia mollior*.

**Figure 4:**
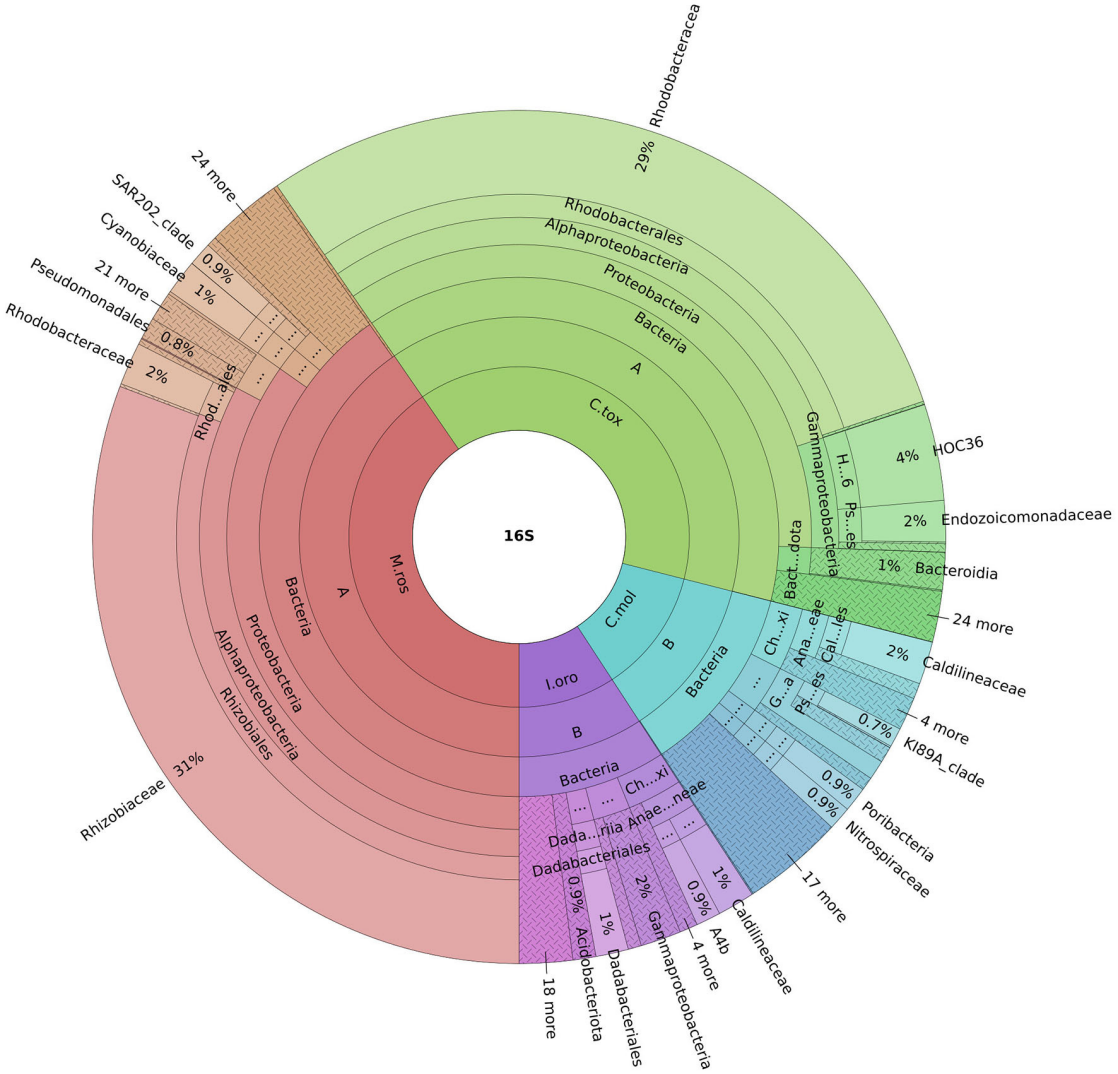
Krona plot represents the most abundant phyla for all sponges. Sample code: M.ros, *M*. (*M*.) *rosacea*; I.oro, *I. oros*; C.mol., *C. mollior*; C.tox., *C*. (*C*.) *toxivaria*.

An important finding concerning the species analyzed in this study concerned the fact that the sponges *M*. (*M*.) *rosacea* and *C*. (*C*.) *toxivaria* collected in the Faro Lake were already recorded in this lake in 2013 suggesting, their presence as persistent (Marra et al., 2016). The sponges collected in the Gulf of Naples are typical species in the Mediterranean Sea. In addition, we investigated the bacterial diversity of the Mediterranean sponges by performing the metataxonomic analysis. Our results revealed that the analyzed sponges hosted various bacterial communities according the sampling site. Interestingly, as reported in Ruocco et al. (2021), sponges collected in the Faro Lake had a more diverse phyla composition than sponges collected in the Gulf of Naples (Figure 4). In addition, PCA analysis revealed interesting results for the sponges collected in Faro Lake. In fact, *M*. (*M*.) *rosacea* is the only sponge with a significant presence of Rhizobiaceae bacteria. The community structure seems to be closely related to the order to which they belong, as demonstrated in the Krona plot and heat map. In fact, *I. oros* and *C. mollior*, belonging to Dictyoceratida order, were recorded as high microbial abundance (HMA) species, while *M*. (*M*.) *rosacea* and *C*. (*C*.) *toxivaria*, belonging to the Poecilosclerida order, were low microbial abundance (LMA) species. HMA sponges harbor a more diverse symbiotic community than LMA, which were found to be extremely stable at seasonal and interannual scales.

This taxonomic analysis was useful for us, being strongly interested in the biotechnological potential of these sponges. In fact, as reported in the introduction, sponges in general are among the marine organisms richest in bioactive compounds of biotechnological interest. There is a strong debate in the literature whether the bioactive compounds isolated from sponges are produced by the sponges themselves, by the organisms associated with them, or by the interaction between sponges and microorganisms. The fact is that the microorganisms associated with sponges play a fundamental role in their production. Many studies reported that sponge-associated bacteria are good candidates for isolating natural compounds with their application in pharmacological, nutraceutical, and cosmeceutical fields. In this context, our study represents an important step in this field, representing one of the first assessments of the biotechnological potential of mentioned sponge-associated bacteria. In all species of sponges are present Alphaproteobacteria and Gammaproteobacteria, which showed antimicrobial activity making them suitable tools for pharmacological purposes (Thiel and Imhoff, 2003; Thakur et al., 2005; Taylor et al., 2007; Werner, 2010; Haber and Ilan, 2014; Brinkmann et al., 2017; Bibi et al., 2020). The phylum Cloreflexi (classes Anaerolineae and Dehaloccoidia) is typical of both collection sites, being in all sponge samples. However, no data had been reported so far for marine biotechnology applications for class Anaerolineae. Despite playing an important role in biochemical cycles in various environments, they are manifestly difficult to cultivate due to slow growth (Nakahara et al., 2019; De Castro-Fernández et al., 2023). On the contrary, the anaerobic Dehalococcoides showed an interesting capability to transform various chlorinated organics, which are normally released through industrial and agricultural activities (Bedard et al., 2007; Taş et al., 2010). In the case of the sponge *M*. (*M*.) *rosacea*, Rhizobiaceae was the most abundant family (order Alphaproteobacteria) among other bacteria isolated. These bacteria are generally associated with soil and plant hosts and are involved in the process of biological nitrogen fixation (Carrareto-Alves et al., 2015). Therefore, the family Rhizobiaceae, described in this study, was found associated with the sponge *M*. (*M*.) *rosacea* for the first time. Potentially, these bacteria can be used for bioremediation of heavy metals and biodegradation of toxic compounds due to their metabolic capabilities and ecological roles (Aoki et al., 2022). However, their role in marine environments remain unclear because of the limited availability of cultured marine isolates and their sequenced genomes (Kimes et al., 2015). In summary, our data drew attention to the biodiversity of species in the Mediterranean Sea and the 16S rRNA sequence dataset, allowing the detection of several resident microbiomes featured. The sponges and their associated microorganisms revealed good source to identify new compounds for biotechnological applications, and further analyses will be needed to investigate the potential role of the bacteria associated to these sponges. In the meantime, we analyzed the biotechnological potential of these sponges by bioassay-guided fractionation on several human cancer lines, and first results showed specific antimitotic activity against some cancers. We also tried identifying the chemical structure of the compounds responsible for this activity. We also performed metagenomic and transcriptomic analyses on these sponges, which could help us identify the gene/enzyme responsible for the production of the bioactive compounds. These –omic techniques represent an environmental-friendly approach mainly for organisms, such as the sponges, which cannot be cultivated and are difficult to keep in the laboratory to increase biomass.

## Data availability statement

The original contributions presented in the study are included in the article/Supplementary material, further inquiries can be directed to the corresponding authors.

## Ethics statement

The manuscript presents research on animals that do not require ethical approval for their study.

## Author contributions

RE: Data curation, Investigation, Methodology, Writing – original draft, Writing – review & editing. SF: Data curation, Methodology, Writing – original draft. MS: Data curation, Formal analysis, Methodology, Writing – review & editing. SR: Data curation, Formal analysis, Methodology, Writing – review & editing. MB: Data curation, Formal analysis, Investigation, Methodology, Writing – review & editing. NR: Data curation, Methodology, Writing – review & editing. GZ: Data curation, Methodology, Writing – review & editing. MGi: Data curation, Methodology, Supervision, Writing – review & editing. MP: Data curation, Methodology, Supervision, Writing – review & editing. MGu: Data curation, Methodology, Writing – review & editing. VZ: Conceptualization, Resources, Writing – original draft, Writing – review & editing. MC: Conceptualization, Funding acquisition, Investigation, Methodology, Resources, Validation, Writing – original draft, Writing – review & editing.
