# Supplementary material for: Characterizing the bacterial communities associated with Mediterranean sponges: a metataxonomic analysis
Source: Front Microbiol. 2024 Jan 11;14:1295459. doi: 10.3389/fmicb.2023.1295459 (PMC10808595; doi:10.3389/fmicb.2023.1295459)
Supplement: Supplementary file 5 [file Data_Sheet_1.docx]

Supplementary Material

**
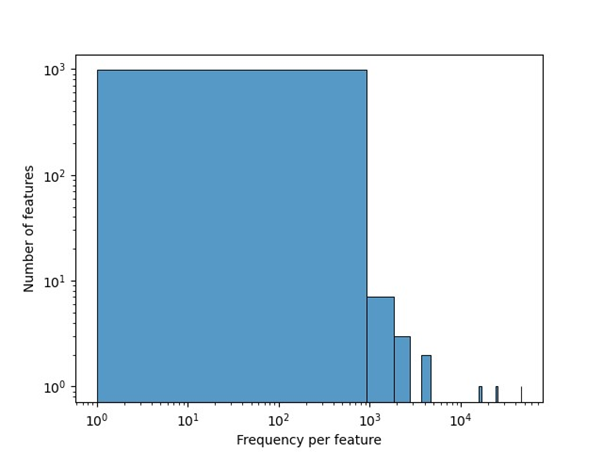
**

**Supplementary Figure 1.** Distribution of OTU’s frequencies.

**
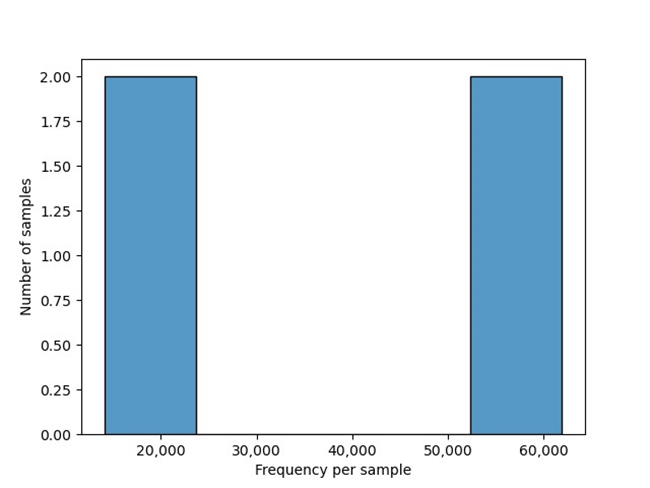
**

**Supplementary Figure 2.** Distribution of OTU’s frequencies for each sample (reported as a blue bar).
